# Development of a spatial skills test for 4th grade primary school students: validity and reliability study

**DOI:** 10.3389/fpsyg.2026.1901482

**Published:** 2026-09-03

**Authors:** Elif Aydın-Çolak, Neslihan Şahin, Mustafa Kasım Kıroğlu

**Affiliations:** 1 Department of Primary Education, Graduate School of Education, Ondokuz Mayıs University, Samsun, Türkiye; 2 Department of Mathematics Education, Faculty of Education, Sinop University, Sinop, Türkiye; 3 Department of Primary Education, Faculty of Education, Ondokuz Mayıs University, Samsun, Türkiye

**Keywords:** primary school students, spatial skills test, spatial orientation, spatial relations, spatial visualization

## Abstract

Although several instruments have been developed to assess spatial skills, most were designed for older students, emphasize specific components of spatial ability, or have not been specifically designed to reflect the developmental characteristics and mathematics curriculum of fourth-grade primary school students. Therefore, this study aimed to develop a valid and reliable spatial skills test specifically designed for fourth-grade primary school students. The study employed a quantitative methodological instrument development design. First, a literature review was conducted to generate an item pool, after which the test’s content validity was established through expert evaluations. The draft test was administered to 360 fourth-grade students attending public primary schools in the Black Sea Region of Türkiye during the 2024–2025 academic year. Item difficulty and discrimination indices, corrected item–total correlations, lower–upper group comparisons, the Kuder–Richardson-20 (KR-20) reliability coefficient, confirmatory factor analysis (CFA), and Rasch model analysis were used to evaluate the instrument’s psychometric properties. The findings resulted in a final 23-item test consisting of three dimensions: spatial visualization, spatial relations, and spatial orientation. The KR-20 coefficient was 0.827, indicating good internal consistency, while the Rasch analysis demonstrated acceptable item fit and satisfactory person separation reliability. Although the CFA provided partial support for the proposed three-dimensional structure, the construct validity evidence was strengthened by the complementary findings obtained from the Rasch model and classical test theory analyses. Overall, the findings indicate that the developed instrument provides promising evidence of validity and reliability for assessing the spatial skills of fourth-grade primary school students.

## 1 Introduction

The concepts of spatial thinking, spatial reasoning, spatial ability, and spatial skills are used interchangeably, yet they differ semantically. To eliminate terminological differences and determine the connection of these terms with the mathematics course, Uttal (2017) defined “spatial thinking” as the most general concept and stated that spatial reasoning includes the concepts of “spatial skill” and “spatial ability.” Spatial thinking is a fundamental cognitive skill that enables us to mentally process the shape, position, and movement of objects, understand spatial information by encoding it through symbolic systems such as language, maps, and diagrams, and share it with others (Uttal et al., 2023). An analytically important distinction also concerns the terms’ *ability* and *skill*. Whereas spatial ability is generally used to describe relatively stable individual differences in spatial performance (Wai et al., 2009), spatial skill emphasizes performance that can be developed through instruction and experience (Uttal, 2017). Because the present study focuses on an educationally relevant construct that can be fostered and assessed, the term *spatial skills* is used when referring to the construct examined in this study.

Many forms of scientific thinking are inherently spatial, and even non-spatial information is often conveyed through maps, diagrams, graphs, analogies, and other forms of spatial communication (Newcombe, 2010). In general, spatial thinking reflects a cognitive capacity that is closely related not only to general intelligence but also, in particular, to the solution of non-routine mathematics problems (Clements and Sarama, 2011). Mathematics is a fundamental area in which spatial reasoning can be explored and developed in depth (Thom et al., 2024). Recent studies have strongly demonstrated the relationship between spatial skills and mathematical achievement (Cui and Guo, 2022; Geer et al., 2019; Gilligan et al., 2019; Johnson et al., 2022; Lowrie et al., 2019, 2020; Lowrie and Logan, 2023; Möhring et al., 2021; Scott et al., 2024; Sorby and Panther, 2020; Xie et al., 2020; Xu et al., 2025; Young et al., 2018). It was determined that children who were successful in spatial tasks tended to exhibit strong mathematical performance (Bailey, 2017; Lowrie et al., 2020). On the other hand, Sinclair and Bruce (2015) found that the lack of development of spatial skills in students negatively affected the development of their mathematical thinking skills. The relationship between spatial ability and mathematics has been demonstrated clearly: the question is no longer whether they are related but how they are connected; that is, the need to understand the causal mechanisms and shared processes has emerged (Mix and Cheng, 2012). Therefore, current research focuses not only on the existence of this relationship but also on the shared processes and mechanisms that connect spatial and mathematical skills.

There is strong evidence that spatial skills can be developed (Cheng and Mix, 2014; Hawes et al., 2022; Newcombe, 2010). These skills develop substantially during infancy and early childhood (Newcombe and Frick, 2010). Practices based on visual cues and bodily movements have been shown to improve young children’s spatial skills (Burte et al., 2017; McCluskey et al., 2023; Yang et al., 2020). Gains resulting from interventions designed to develop spatial skills have also been found to transfer to elementary school mathematics skills (Carr et al., 2018). The National Research Council (2006) similarly states that differences in spatial thinking can be shaped by maturation, education, and experience and that practice can improve performance in constructing and transforming spatial representations. However, it also emphasizes that this development and its transfer to different tasks may depend largely on the context and spatial patterns involved (National Research Council, 2006). The malleability of spatial skills increases the need for valid and reliable measures to monitor their development and evaluate the effects of instructional interventions (Nagy-Kondor, 2024). Therefore, developing assessment instruments appropriate for the characteristics of elementary school students is important both for examining the development of spatial skills and for evaluating instructional practices that support these skills. In this context, the quality of play- and concrete-material-based experiences that support children’s spatial skills should also be considered.

Experiences with spatial toys, such as blocks, puzzles, and shape games, and the spatial words and gestures these evoke in adults have a significant effect on the early development of spatial skills (Verdine et al., 2014). Children can, for example, identify block structures from different viewpoints, match the appearances of the same structure depicted from different viewpoints, or try to find the viewpoint from which a photograph was taken (Clements, 1998). During block building, children use many of these strategies (mental rotation, spatial visualization) to construct whole structures by combining blocks, rotating blocks in different directions, and exploring the spatial relations among blocks (Casey et al., 2008). Previous studies have consistently shown that play can develop spatial skills (Weber et al., 2024). Playing with spatial toys and participating in spatial activities are considered important parts of the development of spatial thinking (Newman et al., 2016). There is evidence that children who play with spatial toys (e.g., puzzles and blocks) show spatial growth (Jirout and Newcombe, 2015; Newman et al., 2016). Ng et al. (2022) used unit cubes (blocks) to develop and concretize the spatial skills of primary school students. Burte et al. (2017) used pop-up papers. Gilligan et al. (2019) used computer-based applications. Studies have shown a positive relationship between block-building skills and mathematics skills (Nath and Szücs, 2014). These findings indicate that the use of blocks constitutes an important and effective learning tool for supporting children’s spatial skills. However, examining the development of these skills and assessing them reliably require a clear identification of the fundamental dimensions that constitute spatial skills and an explicit definition of these dimensions.

### 1.1 Spatial skills and fundamental components

Spatial skills are conceptualized in the literature not as a unidimensional construct but as a multidimensional one. The components most frequently addressed in the psychometric and educational literature are spatial visualization, spatial relations, and spatial orientation (Contero et al., 2005; D'Oliveira, 2004; Guilford and Zimmerman, 1948; McGee, 1979). Although some studies also include additional components such as spatial perception and mental rotation (Linn and Petersen, 1985; Maier, 1998), spatial visualization, spatial relations, and spatial orientation remain among the most frequently examined dimensions in the psychometric and educational literature.

Spatial visualization, a subdimension of spatial skill (Alias et al., 2002), encompasses mentally imagining movements, transformations, and other changes in visual objects (Guilford and Zimmerman, 1948); forming mental images of objects and spatial figures (Contero et al., 2005); and mentally rotating, manipulating, and transforming two- or three-dimensional objects (McGee, 1979). This skill also involves imagining how objects appear from different perspectives, visualizing two-dimensional representations of three-dimensional objects, and mentally decomposing and recombining shapes (Battista, 2004). The common feature of these definitions is the construction of mental representations of two- and three-dimensional objects and the ability to process these representations mentally through movement, transformation, changes in perspective, decomposition, and recombination.

Spatial relations encompass mentally visualizing the effects of operations such as rotation, reflection, and inversion on objects (Barnea, 2000), mentally rotating two-dimensional objects (Contero et al., 2005), and understanding the spatial arrangement of objects or object parts and the relationships among them (Maier, 1998). This skill also includes solving simple rotation problems and recognizing rotated or reflected forms of a target figure (D'Oliveira, 2004). The common feature of these definitions is the ability to understand the spatial arrangement of objects or object parts relative to one another and to mentally determine how this arrangement would appear following transformations such as rotation, reflection, or inversion.

Spatial orientation encompasses understanding one’s position relative to objects in space and navigating based on these relationships (Clements, 1998), mentally visualizing how an object or spatial arrangement would appear from a different perspective (Barnea, 2000; Contero et al., 2005; Lohman, 1979), and accurately perceiving a spatial arrangement regardless of the direction from which it is presented and relating it to one’s own body (McGee, 1979). The common feature of these definitions is the ability to use one’s own position as a reference point to accurately perceive relationships among objects and spatial arrangements from different perspectives and mentally reconstruct them.

These definitional differences arise not so much from directly conflicting theoretical approaches as from differences in research purposes, participant characteristics, the tasks and assessment instruments employed, and analytical approaches. While some studies focus on the psychometric classification of spatial skills, others aim to explain specific cognitive processes or performance in educational contexts. Moreover, because tasks such as rotation, reflection, mental transformation, and perspective taking may require multiple spatial processes simultaneously, the definitional boundaries among these dimensions may partially overlap. Therefore, spatial visualization, spatial relations, and spatial orientation should be regarded not as mutually exclusive categories but as dimensions that emphasize related yet distinct aspects of spatial skills.

Accordingly, in this study, the conceptual scope of spatial skills was limited to the dimensions of spatial visualization, spatial relations, and spatial orientation, which are frequently addressed in the literature. In developing the assessment instrument, the cognitive processes common to the various definitions of each dimension were taken into account. Accordingly, spatial visualization was conceptualized as the formation of mental representations of two- and three-dimensional objects and the mental processing of these representations through movement, transformation, changes in perspective, decomposition, and recombination; spatial relations as understanding the spatial arrangement of objects or object parts relative to one another and mentally determining how this arrangement would appear following rotation, reflection, or inversion; and spatial orientation as perceiving objects and spatial arrangements from different perspectives and mentally reconstructing them by using one’s own position as a reference point. The dimensions of the spatial skills test were delimited within the framework of these shared definitions.

### 1.2 Context

Spatial skills are essential for students (Herawati and Hariyani, 2024; Pinilla, 2024) because well-developed spatial skills support a good understanding of mathematics (Bates et al., 2023; Herawati and Hariyani, 2024; Lowrie and Logan, 2023).

In the literature on defining spatial ability, inconsistent definitions, differing numbers and names of factors, and tests measuring these factors both among authors and within the same authors’ studies create conceptual ambiguity in the field (Bates et al., 2023; D'Oliveira, 2004). To develop cognitive skills, especially spatial intelligence, we must be able to measure them (Nagy-Kondor, 2024). Many examples of spatial skill tests are seen in the relevant literature (Ekstrom et al., 1976; Guay, 1977; Guilford and Zimmerman, 1948; Hegarty and Waller, 2004; Kozhevnikov and Hegarty, 2001; Shepard and Metzler, 1971; Thurstone, 1943; Witkin, 1950). Differences in the definition of spatial ability become even more complex due to differences in the methods and measurement instruments researchers use to evaluate different dimensions of spatial skills (Eliot and Hauptman, 1981). These confusions have also affected the results of factor analysis studies conducted to determine test structures (Açıkgül-Fırat and Köksal, 2018).

Although numerous spatial ability tests have been developed, several important limitations remain. Existing instruments differ substantially in their theoretical foundations, factor structures, target age groups, and psychometric quality. Many were originally designed for adolescents or adults, while relatively few have been specifically developed and validated for primary school children. Moreover, currently available instruments often focus on only one component of spatial ability and may not adequately capture the multidimensional nature of spatial skills within mathematics education. As a result, important questions remain unresolved regarding how spatial skills should be operationalized for young learners, which dimensions should be assessed simultaneously, and how a psychometrically sound instrument can be developed for use within the context of elementary mathematics education.

Uttal et al. (2023) stated that spatial skill measurement instruments carry significant limitations due to accessibility, psychometric adequacy, the constructs they cover, and age-group restrictions. Considering the diversity of findings in the literature, a necessary step toward understanding spatial ability is the reorganization of psychometric data for standardization (Pellegrino et al., 1984). In this case, the importance of developing tests in accordance with the characteristics of a specific group and testing their psychometric properties can be acknowledged. It has been argued that spatial skills can be improved when appropriate educational environments are provided (Bufasi et al., 2024; Yang et al., 2020; Hawes et al., 2022). Although there are studies on the development of spatial skills, fewer studies are encountered regarding primary school students (Alkouri, 2022; Hasanah et al., 2024).

Research on spatial ability in Türkiye has generally focused on geometric solids and volume measurement, perspective, and mental rotation, and as measurement instruments, mostly the Purdue Spatial Visualization Test developed by Guay (1977) and the MGMP Spatial Ability Test developed within the Middle Grades Mathematics Project by faculty members of the Mathematics Department at Michigan State University (1983) have been used. To develop the test, the factors of spatial visualization, spatial relations, and spatial orientation were first defined based on the literature. While creating *spatial visualization* questions, Guilford and Zimmerman’s (1948) definition of imagining movements, transformations, or other changes in visual objects was taken as a reference. Barnea’s (2000) definition of mentally imagining the effects of operations such as rotation, reflection, and inversion, or the ability to manipulate objects mentally, was used as a basis for creating spatial relations questions. Contero et al.’s (2005) definition of individuals’ ability to imagine how objects will appear from a different viewpoint was used to create spatial orientation questions.

Existing spatial ability tests, such as the Purdue Spatial Visualization Test and the MGMP Spatial Ability Test, have made important contributions to spatial cognition research. However, these instruments were not specifically designed for fourth-grade primary school students within the context of mathematics education. In addition, they differ considerably in their conceptual definitions, factor structures, and item characteristics, making it difficult to obtain consistent measurements across studies. These limitations highlight the need for a developmentally appropriate, psychometrically validated instrument that simultaneously assesses multiple dimensions of spatial skills in young learners.

In this context, accurately measuring the development of spatial skills among students and revealing the effects of education will only be possible by using a valid and reliable spatial skill test. Consequently, this study aimed to develop a spatial skill test for fourth-grade primary school students in the context of the mathematics course, in which the definitions of spatial ability factors and question types were described in detail and statistically tested. The present study addresses these limitations by developing a spatial skills test specifically designed for fourth-grade primary school students. Unlike many existing instruments, the proposed test integrates three theoretically supported dimensions—spatial visualization, spatial relations, and spatial orientation—within a single assessment framework. Furthermore, all items are based on unit-cube constructions that closely align with elementary mathematics learning activities, thereby providing greater content relevance for classroom assessment. The study also establishes the instrument’s psychometric properties through comprehensive item analyses, reliability estimation, and confirmatory factor analysis. The questions on the test were created using unit-cube (block-based) designs because block building is a common play activity among young children and is widely used in spatial cognition research to assess three-dimensional spatial reasoning (Zhang et al., 2020). Beyond their familiarity, unit cube representations provide a standardized three-dimensional visual context that enables the assessment of spatial visualization, spatial relations, and spatial orientation within a common item format while minimizing the influence of reading comprehension and linguistic demands. Therefore, unit cubes were intentionally selected to ensure developmental appropriateness and measurement consistency for fourth-grade primary school students. The test developed in this study focused on the three factors of spatial ability (spatial visualization, spatial relations, and spatial orientation). Each factor was created using different question types to broaden the test’s scope.

Accordingly, this study aims to develop and validate a multidimensional spatial skills test for fourth-grade primary school students. By addressing conceptual inconsistencies, focusing on a developmentally appropriate population, and providing comprehensive psychometric evidence, the proposed instrument is expected to contribute both to mathematics education research and to classroom assessment practices.

## 2 Method

### 2.1 Design

This study employed a quantitative methodological research design focusing on instrument development. Methodological research aims to develop measurement instruments and evaluate their psychometric properties (DeVellis, 2016). Accordingly, the present study sought to develop a valid and reliable Spatial Skills Test for fourth-grade primary school students by examining its content validity, item difficulty, item discrimination, corrected item–total correlations, lower–upper group differences, internal consistency (KR-20), confirmatory factor analysis (CFA), and Rasch model analysis.

### 2.2 Implementation

The study began with the creation of an original item pool for a spatial skills test. For this purpose, the relevant literature was reviewed, and a large number of original items to measure the fundamental dimensions of spatial skills were designed. In this context, the 35-item pool initially created in line with the literature was submitted for expert review. After the test’s content validity was ensured, the pilot implementation was conducted. The items prepared for the item pool were submitted for the opinions of 7 field experts, including 1 language expert, 1 measurement and evaluation expert, 2 mathematics education experts, 2 primary education experts, and 1 visual arts expert, to be evaluated in terms of content and structure. Based on the expert opinions, the feedback was grouped under the headings of comprehensibility, changes in the question stem, use of common concepts, page layout, distractor level, difficulty level of the questions, and similarities among questions. To establish content validity, the initial pool of 35 items was reviewed by seven experts in mathematics education, elementary education, educational measurement and evaluation, and spatial ability. Each item was evaluated for alignment with the intended learning outcome, clarity and comprehensibility, developmental appropriateness, time appropriateness, and the quality of distractors. Based on the experts’ written feedback and recommendations, necessary revisions were made to the wording, visual presentation, and distractors of the items before the final version of the test was established.

**Figure 1:**
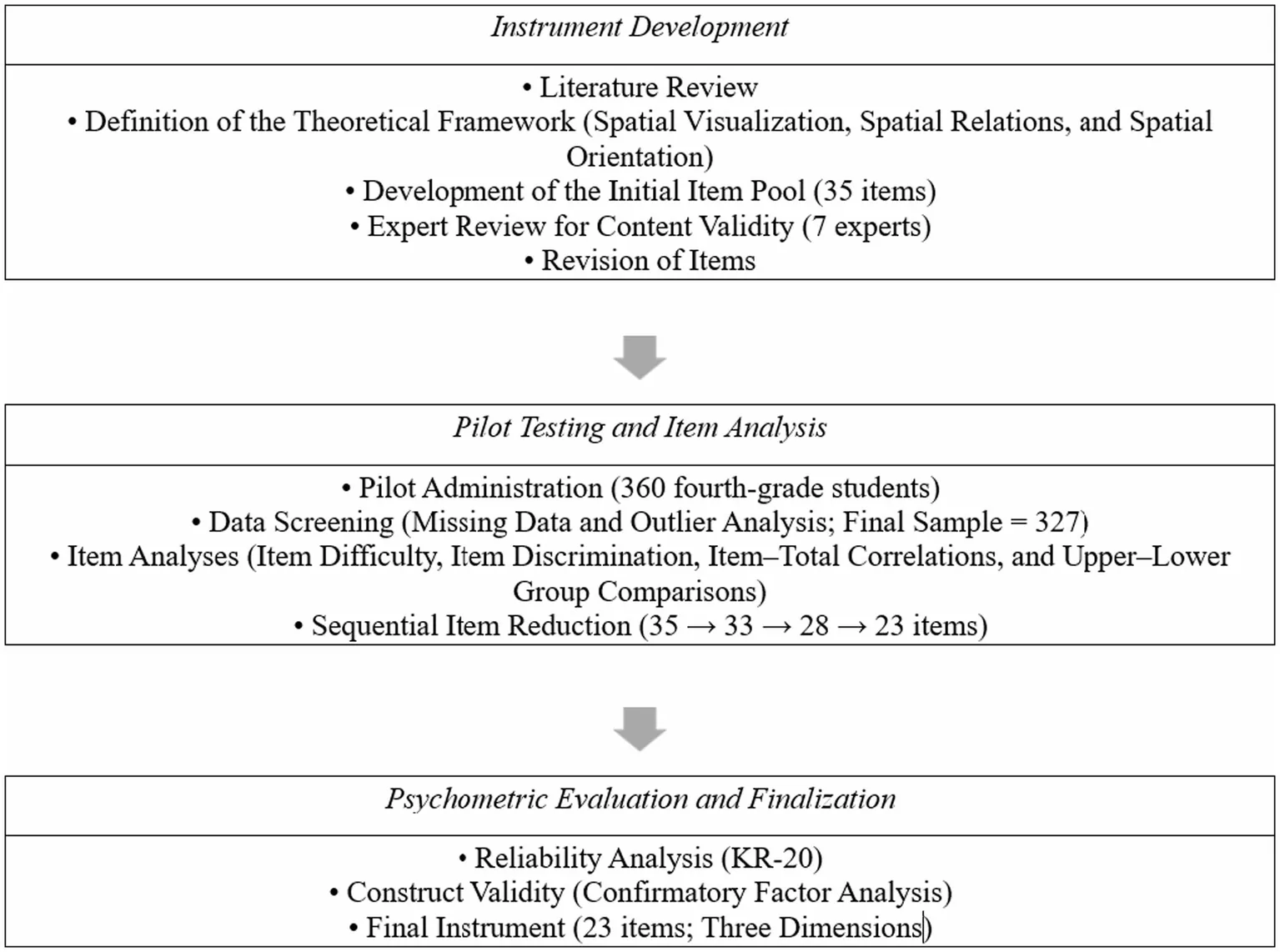
Instrument development process.

The instrument development process was carried out through a series of sequential methodological stages. Figure 1 presents an overview of the procedures followed from item generation to the construction of the final 23-item instrument.

As illustrated in Figure 1, the development of the Spatial Skills Test followed a systematic and sequential process, beginning with item generation and expert evaluation and concluding with psychometric validation and the construction of the final 23-item instrument. This process ensured that each stage informed the subsequent refinement of the instrument.

The analysis process first began with checks for missing data, outliers, and suitability. Then, the Item Difficulty Index (Pj) and Item Discrimination Index (Rjx) were calculated, items showing low performance were identified, and some items were removed from the test based on both statistical and conceptual justifications. Afterward, the item structure was examined using the mean differences between lower and upper groups, the correlation-based item analysis, and the item-total score correlations. The reliability of the test was calculated using the Kuder–Richardson-20 (KR-20) coefficient, and the resulting value of 0.827 indicated high internal consistency. As a result of all these analyses, a valid and reliable 23-item skill test form that carried sufficient characteristics in terms of both statistics and measurement theory was created.

### 2.3 Participants

The test was administered to 360 fourth-grade primary school students enrolled in schools in a province in the Black Sea Region of Türkiye during the spring semester of the 2024–2025 academic year. The study procedures were conducted in classroom settings in approximately 60-min sessions under the guidance of the researcher and form teachers. The dataset was checked for missing responses and inconsistencies, and the responses of 24 students who did not meet these criteria were removed from the test. In the analysis, data from 336 students were entered into the system; however, the response forms of 9 students who were statistically identified as outliers were removed, and the analyses proceeded with data from 327 students.

Initially, data were collected from 360 fourth-grade students. During the preliminary data screening, 24 response forms were excluded because they contained incomplete or invalid responses, resulting in a dataset of 336 participants. Subsequently, boxplot analysis was performed to identify univariate outliers, and 9 additional cases were excluded for exhibiting atypical response patterns. Consequently, the final psychometric analyses were conducted using data from 327 participants.

The target sample size was determined based on widely accepted methodological recommendations for instrument development and confirmatory factor analysis rather than through an *a priori* statistical power analysis. Methodological guidelines recommend a minimum sample of approximately 200 participants for confirmatory factor analysis and approximately 5–10 participants per item in instrument development studies. Since the initial item pool consisted of 35 items, recruiting 360 participants was considered sufficient to ensure stable parameter estimation and robust psychometric analyses. Following data screening and the exclusion of incomplete responses and multivariate outliers, the final analyses were conducted using data from 327 participants, which also exceeded these commonly recommended sample size criteria (DeVellis and Thorpe, 2021; Kline, 2023; Worthington and Whittaker, 2006).

It was observed that 172 participants were girls (52.6%) and 155 were boys (47.4%). This distribution shows that the sample was balanced in terms of gender. No missing data were received from the final sample of students (327 students), and the data of all participants were included in the analysis process.

Data screening was conducted in two consecutive stages. First, boxplot analysis was used to identify extreme observations, resulting in the exclusion of 24 participants. Subsequently, the remaining dataset was examined for incomplete or unsuitable response patterns, and nine additional cases were removed. Consequently, all psychometric analyses were conducted using data obtained from 327 participants.

### 2.4 Data collection

Data from the Spatial Skill Test were collected from 360 fourth-grade primary school students in a province of the Black Sea Region of Türkiye during the spring semester of the 2024–2025 academic year. The necessary permissions were obtained to collect the data, and the study was conducted with voluntary participants between 2 June 2025 and 18 June 2025. Twelve schools were included in the study during data collection. While determining these schools, the data of the Socio-Economic Development Ranking Studies (Ministry of Industry and Technology of the Republic of Türkiye, 2022), which analyzes the demographic, employment, and social security, education, health, finance, competitiveness, innovativeness, and quality of life variables of districts, were used. In this context, schools were selected from districts with varying levels of socio-economic development to ensure data diversity. During implementation, standard instructions were provided to all participants, and efforts were made to ensure equal conditions. The test implementation was carried out under the guidance of form teachers and the researcher so that participants could see themselves as important stakeholders in the process and reflect their learning more accurately. In this process, the researcher observed how the participants solved the questions, their behaviors, and how they used their time. Based on the observations, the test implementation duration was determined to be an average of 50 min.

### 2.5 Data analysis

The data obtained from the Spatial Skills Test in the study underwent comprehensive preprocessing before analysis. First, the dataset was examined for missing data, and it was determined that all participants’ responses were complete. Then, an outlier analysis was conducted, and it was determined that all z-scores were within ±3, and no observations were excluded from the dataset. With this procedure, the possibility of outliers affecting the reliability of the analyses was eliminated (Field, 2018).

Since the data’s scoring structure was dichotomous (0 = incorrect, 1 = correct), normality assumptions were not assessed. In this context, the distribution characteristics of the test scores were evaluated using descriptive statistics. For each item, mean, standard deviation, and frequency were calculated, and the overall distribution was revealed.

In the item-level analyses, the Item Difficulty Index (Pj) and Item Discrimination Index (Rjx) for each test item were first calculated. Using these analyses, the extent to which the items were discriminative and how easy or difficult they were for participants was evaluated. The item analysis based on the mean difference of the lower-upper groups was then conducted, and whether each item distinguished individuals with low and high achievement levels was determined. For this analysis, the sample was divided into three groups, and the mean differences of the 27% lower and upper groups were calculated and interpreted (Kelley, 1939; Nitko and Brookhart, 2014).

The internal consistency of the items was examined using correlation-based item analyses, and the magnitude of each item’s relationship with the overall test was evaluated. In this regard, items with Corrected Item-Total Correlations below 0.20 were considered to have low discrimination (Tavşancıl, 2014).

After all these item-level analyses, the test’s reliability was calculated using the Kuder–Richardson-20 (KR-20) coefficient, which is appropriate for dichotomous items. The KR-20 value was found as 0.827, and since this value was above 0.70, it was concluded that the internal consistency of the test was high (Kuder and Richardson, 1937). As a result of these procedures, a test form consisting of 23 items with a valid and reliable item structure was obtained.

All classical item analyses, including item difficulty, item discrimination, lower–upper group comparisons, item–total correlations, and KR-20 reliability analyses, were conducted using IBM SPSS Statistics 27 (IBM Corp., Armonk, NY, USA). Confirmatory factor analysis was performed using the Confirmatory Factor Analysis module in Jamovi (Version 2.6), which is based on the *lavaan* package in R. Because the Spatial Skills Test consists of dichotomously scored achievement items and was developed within the framework of Classical Test Theory, the Kuder–Richardson-20 (KR-20) coefficient was used as the primary indicator of internal consistency. Although Composite Reliability (CR) is widely reported for latent variable models, KR-20 is the recommended reliability coefficient for dichotomously scored achievement tests. Therefore, the reliability of the present instrument was primarily evaluated using the KR-20, along with item difficulty, item discrimination, corrected item–total correlations, and lower–upper group comparisons.

In addition to classical test theory analyses and confirmatory factor analysis, a Rasch model analysis was conducted using the dichotomous Rasch model to obtain complementary evidence on the instrument’s psychometric properties. The Rasch analysis was performed in Jamovi (Version 2.6) using the snowRMM module. Item fit was evaluated using Infit and Outfit mean-square (MNSQ) statistics, with values between 0.50 and 1.50 considered acceptable. In addition, person separation reliability and the Wright Map were examined to evaluate measurement precision and the correspondence between item difficulty and participant ability. These analyses provided complementary evidence supporting the measurement quality of the developed instrument beyond the classical test theory analyses (Bond et al., 2021; Linacre, 2024).

## 3 Results

### 3.1 Content validity and preparation of the data for analysis

The content validity of the test was analyzed to determine the extent to which the cognitive skills intended to be measured were represented by the items. In this regard, the theoretical constructs and literature on spatial skills were examined, and an item pool appropriate to the cognitive objectives was created. The items were structured to correspond to the dimensions and skill areas. Each item was written based on observable, measurable behaviors, and Bloom’s Cognitive Domain Taxonomy and theoretical frameworks of spatial thinking were taken into consideration (Anderson and Krathwohl, 2001). The item pool was submitted to seven experts for their opinions, and the appropriateness of the items for the skills in question and the adequacy of the test content were evaluated. In line with the feedback, simplifications and visual improvements were made to some items. Most of the experts found the representational power of the items sufficient. This process established the scientific foundation that would support the content validity of the measurement instrument (Haynes et al., 1995).

There were no missing values in the dataset, so no additional procedures were performed. Outliers were examined using z-score (±3) and boxplot analyses, and a total of 9 observations were removed (Field, 2018; Tabachnick and Fidell, 2019). Analyses were conducted on the data of 327 participants. Because the instrument consisted of dichotomously scored items, formal assumptions of normality were not considered a prerequisite for the subsequent psychometric analyses.

Spatial Skills Test consisted of dichotomously scored items (0 = incorrect, 1 = correct); item-level normality assumptions were not considered a prerequisite for the psychometric analyses conducted within the framework of Classical Test Theory or the Rasch model. Therefore, the distribution of the total test scores was examined descriptively rather than relying on formal normality tests. The total scores showed slight negative skewness (−0.224) and kurtosis (−1.023), indicating no substantial departure from symmetry.

### 3.2 Descriptive statistics

Before examining the structural characteristics of a measurement instrument, it is important to evaluate the descriptive statistics related to the items. The mean, standard deviation, skewness, and kurtosis values provide information about the difficulty level, discrimination, and distribution structure of the items. These values also guide the assessment of normal distribution and the selection of methods to be used in subsequent analyses. For this reason, the descriptive statistics of all items in the test were examined.

As shown in Table 1, the mean scores for the items ranged from 0.38 to 1.00. Some items were easy (e.g., İ4, G8), while others were challenging (e.g., İ5, Y5, Y6, Y7). The mean score of 1.00 for item İ4 indicated that all participants answered correctly, and the discriminatory power of the item could be low. The standard deviations were generally low (0.134–0.501), but they were higher in items such as İ10, İ5, and Y6. The skewness values were mostly negative (between −0.006 and −7.211), which indicated high correct response rates, while the positive skewness values found for İ5, Y5, and Y6 indicated their difficulty. The kurtosis values ranged between −2.012 and 50.303, and the extremely positive value in G8 indicated a substantial deviation from normal distribution. Although the construct appeared consistent overall, some items, such as G8, İ4, Y5, and Y6, were re-evaluated through detailed item analysis.

**Table 1:** Descriptive statistics of all items.

Item	Mean	Standard deviation	Skewness	Kurtosis
G1	0.83	0.375	−1.782	1.184
G2	0.95	0.216	−4.201	15.747
G3	0.78	0.417	−1.335	−0.218
G4	0.63	0.484	−0.527	−1.733
G5	0.69	0.463	−0.831	−1.317
G6	0.83	0.372	−1.812	1.291
G7	0.89	0.313	−2.503	4.291
G8	0.98	0.134	−7.211	5.303
G9	0.75	0.434	−1.155	−0.669
G10	0.76	0.427	−1.233	−0.484
G11	0.73	0.446	−1.028	−0.948
İ1	0.88	0.328	−2.316	3.384
İ2	0.79	0.406	−1.446	0.091
İ3	0.93	0.261	−3.287	8.857
İ4	1.00	0.000	−1.028	−0.948
İ5	0.73	0.487	0.487	−1.774
İ6	0.63	0.484	−0.541	−1.718
İ7	0.86	0.345	−2.114	2.492
İ8	0.47	0.500	0.105	−2.001
İ9	0.86	0.351	−2.040	2.177
İ10	0.50	0.501	−0.006	−2.012
İ11	0.87	0.342	−2.152	2.646
İ12	0.71	0.455	−0.927	−1.148
Y1	0.60	0.491	−0.394	−1.856
Y2	0.69	0.463	−0.831	−1.317
Y3	0.77	0.423	−1.273	−0.382
Y4	0.77	0.423	−1.273	−0.382
Y5	0.39	0.489	0.434	−1.823
Y6	0.41	0.493	0.369	−1.876
Y7	0.38	0.487	−0.487	−1.774
Y8	0.67	0.470	−0.740	−1.462
Y9	0.75	0.436	−1.137	−0.713
Y10	0.42	0.494	0.330	−1.903
Y11	0.80	0.397	−1.541	−1.377
Y12	0.76	0.427	−1.233	−0.484

G, Spatial Visualization; İ, Spatial Relations; Y, Spatial Orientation.

### 3.3 Item analyses

Item analysis is a fundamental technique for evaluating the measurement power of individual items on a test. In this analysis, both the difficulty level (p-index) and the discriminatory power of each item are examined. While the difficulty level determines how easy or difficult an item is, discrimination shows the extent to which the item can distinguish individuals with high and low achievement levels. Discrimination is generally determined by the item-total score correlation coefficient or the difference between the mean correct response rates of the lower 27% and upper 27% groups. Items with high discriminatory power enhance the test’s validity, whereas items with low or negative correlations may compromise the test’s overall structure and are recommended for removal. For this reason, item analyses determine which test items will be retained, improved, or removed (Crocker and Algina, 2006; Büyüköztürk, 2022).

#### 3.3.1 Item difficulty index (Pj)

The item difficulty index is a fundamental measure that indicates the extent to which a test item is answered correctly by participants and is generally denoted by Pj. This value, which ranges from 0 to 1, is calculated as the ratio of participants who answered the item correctly to the total number of participants (p = D/N). A high Pj value indicates that the item is easy, while a low value indicates that it is difficult. In this context, *p* < 0.30 is evaluated as a difficult item, 0.30 ≤ *p* ≤ 0.80 is evaluated as a medium-difficulty item, and *p* > 0.80 is evaluated as an easy item. The difficulty index plays an important role in assessing whether the overall structure of the test is balanced, because a test consisting solely of very easy or very difficult items may be insufficient to distinguish the true performance levels of different individuals (Crocker and Algina, 2006; Büyüköztürk, 2022; Muijs, 2010).

#### 3.3.2 Item discrimination index (Rjx)

The item discrimination index is a statistical indicator that shows the extent to which a test item is discriminative according to the overall achievement levels of individuals and is generally expressed with the symbol Rjx. This index represents the correlation coefficient between the score of each item and the total score of the test. When the Rjx value is positive and high, high-scoring individuals answer the related item more correctly; therefore, the item reflects the whole test and functions as a discriminative item. An Rjx value of 0.30 and above indicates that the item is sufficiently discriminative, whereas values of 0.40 and above indicate strong discrimination. In contrast, values below 0.20 suggest that the item has low discriminatory power and should be reviewed. This index is particularly critical for the validity and measurement power of a test (Büyüköztürk, 2022; Crocker and Algina, 2006).

#### 3.3.3 Evaluation of item difficulty and discrimination indices

In Table 2, the item difficulty index (P_j_) represents the correct response rate for each item, which ranges from 0 to 1. The values of the index being close to 0.50 indicate that the item has discriminative power, while values below 0.20 indicate that the item is too difficult (Turgut and Baykul, 2015). According to the analysis in this study, the difficulty levels of the examined items ranged between 0.38 and 0.98. This situation showed that the test included both easy and moderately challenging items. The great majority of items in the measurement instrument fell within the 0.40 to 0.80 range, indicating that the test had a balanced difficulty level overall. This variety increased the capacity of the measurement instrument to distinguish individuals at different proficiency levels.

**Table 2:** Item analysis results (for 35 items).

Item	Item difficulty index (P_j_)	Item discrimination index (R_jx_)
G1	0.83	0.315**
*G2	0.95	0.099
G3	0.78	0.241**
G4	0.63	0.285**
G5	0.69	0.335**
G6	0.83	0.365**
G7	0.89	0.152**
*G8	0.98	0.106
G9	0.75	0.457**
G10	0.76	0.339**
G11	0.73	0.467**
İ1	0.88	0.232**
İ2	0.79	0.275**
İ3	0.93	0.177**
İ4	0.73	0.319**
İ5	0.38	0.402**
İ6	0.63	0.570**
İ7	0.86	0.365**
İ8	0.47	0.396**
İ9	0.86	0.222**
İ10	0.50	0.496**
İ11	0.87	0.303**
İ12	0.71	0.416**
Y1	0.60	0.484**
Y2	0.69	0.569**
Y3	0.77	0.570**
Y4	0.77	0.494**
Y5	0.39	0.627**
Y6	0.41	0.590**
Y7	0.38	0.514**
Y8	0.67	0.341**
Y9	0.75	0.587**
Y10	0.42	0.230**
Y11	0.80	0.439**
Y12	0.76	0.525**

*Items removed from the test.

The item discrimination index (R_jx_) measures the correlation between each item and the total test score and is used to evaluate the item’s discriminative power. This value is considered good if it is 0.30 and above, moderate if it is between 0.20 and 0.29, and weak if it is 0.19 and below (Büyüköztürk, 2022; Crocker and Algina, 2006). As shown in Table 2, a significant proportion of the 35 items examined in the relevant analyses had discrimination coefficients above 0.30. In particular, items Y1, Y2, Y3, Y4, Y5, and Y6 presented quite strong discriminative characteristics with their high correlation coefficients above 0.50. However, it is seen that the discrimination indices of some items, such as G2 and G8, were low (r < 0.20). This situation indicated that these items should be reviewed, or if necessary, removed from the test. Overall, the measurement instrument was satisfactory in terms of item discrimination.

Considering the item difficulty index (P_j_) and the item discrimination index (R_jx_) together in the item analysis, it is evident that some items need to be removed from the measurement instrument. Very easy (P_j_ > 0.90) or very difficult (P_j_ < 0.20) items reduce the discrimination of the test, while items with low discrimination coefficients (R_jx_ < 0.20) negatively affect reliability because they cannot identify sufficient differences among individuals (Büyüköztürk, 2022; Turgut and Baykul, 2015; Crocker and Algina, 2006). According to these criteria, the P_j_ value of item G2 was found as 0.95, and its R_jx_ value was 0.099, while the P_j_ value of item G8 was found as 0.98, and its R_jx_ value was 0.106. Since these two items were both too easy and did not show significant discrimination among the participants, they were among the items that needed to be removed to ensure the validity and reliability of the measurement instrument.

#### 3.3.4 Item analysis based on the mean difference of the lower-upper groups

Item analysis based on the mean difference of the lower-upper groups is a method based on classical test theory used to determine the discriminatory power of test items. In this analysis, participants are generally divided into two groups of 27% who receive the highest and lowest scores from the test (Kelley, 1939). The mean correct response rates for each item across these two groups are compared, and the item’s discriminatory power is evaluated. The upper group mean being significantly higher than the lower group mean shows that the item can distinguish successful individuals from unsuccessful ones (Turgut and Baykul, 2015; Büyüköztürk, 2022). The obtained difference values provide additional information about item quality and allow stronger inferences about the item when used together with the item discrimination index. The comparison of the mean values of the lower and upper groups is presented in Table 3.

**Table 3:** Comparison of upper-lower group mean values (for 33 items).

Item	Lower group mean	Upper group mean	Diff. (Upper - Lower)
G1	0.66	0.97	0.31
G3	0.65	0.95	0.30
G4	0.46	0.77	0.31
G5	0.52	0.86	0.34
*G6	0.64	0.86	0.22
G7	0.82	0.95	0.13
G9	0.49	0.98	0.49
G10	0.61	0.98	0.37
G11	0.49	0.98	0.49
İ1	0.65	0.92	0.27
İ2	0.52	0.92	0.40
*İ3	0.85	0.98	0.13
İ4	0.52	0.92	0.40
İ5	0.27	0.73	0.46
İ6	0.27	0.73	0.46
İ7	0.69	0.98	0.29
İ8	0.27	0.86	0.59
İ9	0.75	0.97	0.22
İ10	0.20	0.89	0.69
*İ11	0.75	0.97	0.22
İ12	0.48	0.97	0.49
Y1	0.35	0.92	0.57
Y2	0.48	0.97	0.49
*Y3	0.43	0.98	0.55
Y4	0.48	1.00	0.52
Y5	0.09	0.92	0.83
Y6	0.11	0.88	0.77
Y7	0.11	0.78	0.67
Y8	0.52	0.86	0.34
Y9	0.40	1.00	0.60
Y10	0.31	0.56	0.25
*Y11	0.57	0.98	0.41
Y12	0.50	1.00	0.50

*Items removed from the test.

As seen in Table 3, based on the item analysis conducted on the differences between the mean scores of the lower and upper groups on the test, some items needed to be removed from the measurement instrument. In this method, the mean score difference between individuals with high achievement levels and individuals with low achievement levels is taken as the basis, and this difference being 0.40 and above indicates that an item has high discriminatory power (Büyüköztürk, 2022; Turgut and Baykul, 2015). Items with a difference value below 0.20 show low discrimination. In this context, items G6 (−0.04), İ3 (0.03), İ11 (0.07), Y3 (0.08), and Y11 (0.10) could be removed from the measurement instrument because their discriminative power was insufficient. The finding that item G6 had a negative difference value indicated that individuals with high achievement levels answered this correctly at lower rates than those with low achievement did. Therefore, this item was considered to potentially harm the validity of the measurement instrument. The remaining analyses were conducted on 28 items.

#### 3.3.5 Item analysis based on correlations

Item analysis based on correlations is a method that evaluates the contribution level of items to the measurement instrument by examining the relationship of the score of each item with the total score of the scale. In this analysis, the corrected item-total correlation is usually calculated, and this value reflects the relationship of the item with the total score after excluding the item’s own score. Correlation coefficients of 0.30 and above show that the item contributes sufficiently to the measurement instrument, while values below 0.20 indicate that the item has low discriminative power and may need to be removed from the instrument (Büyüköztürk, 2022; Tavşancıl, 2014; DeVellis, 2016). This method is frequently used, especially in determining item quality in scales with high internal consistency and a homogeneous structure. The item-total correlation coefficients obtained for the 28 items examined in this study are presented in Table 4.

**Table 4:** Item-total correlation coefficients (for 28 items).

Item	Scale mean if item deleted	Scale variance if item deleted	Corrected item-total correlation	Cronbach’s alpha if item deleted
G1	17.88	25.126	0.254	0.816
*G3	17.93	25.321	0.173	0.819
G4	18.08	24.959	0.212	0.819
G5	18.02	24.754	0.272	0.816
*G7	17.82	25.805	0.099	0.821
G9	17.96	24.339	0.395	0.811
G10	17.95	24.933	0.259	0.816
G11	17.98	24.263	0.4	0.811
*İ1	17.83	25.643	0.141	0.82
İ2	17.92	25.186	0.213	0.818
İ4	17.98	24.889	0.254	0.817
İ5	18.33	24.405	0.328	0.814
İ6	18.08	23.65	0.496	0.807
İ7	17.85	25.069	0.299	0.815
İ8	18.24	24.352	0.328	0.814
*İ9	17.85	25.549	0.154	0.819
İ10	18.21	23.908	0.421	0.81
İ12	18.0	24.448	0.348	0.813
Y1	18.11	23.966	0.418	0.81
Y2	18.02	23.76	0.498	0.807
Y4	17.94	24.294	0.419	0.81
Y5	18.31	23.241	0.58	0.803
Y6	18.3	23.413	0.538	0.805
Y7	18.33	23.804	0.459	0.808
Y8	18.04	24.753	0.266	0.816
Y9	17.96	23.882	0.504	0.807
*Y10	18.29	25.28	0.14	0.822
Y12	17.95	24.215	0.434	0.81

*Items removed from the test.

As shown in Table 4, in the analysis based on item-total score correlations, the items falling below the threshold of 0.30, in particular, did not contribute to the integrity of the scale (Büyüköztürk, 2022; Tavşancıl, 2014). In this context, items G7 (0.099), G3 (0.173), İ1 (0.141), İ9 (0.154), and Y10 (0.140) have statistically low discriminatory power as their corrected item-total correlation coefficients fell below 0.30. Particularly, items G7 and Y10 increased the Cronbach’s alpha coefficient of the test when deleted, and this situation showed that these items negatively affected the internal consistency of the test. For this reason, it was advisable to remove these items from the test to increase the reliability and item homogeneity of the measurement instrument.

The item elimination procedures continued by taking into account the item difficulty and discrimination indices, the lower and upper group mean differences, and the item-total correlations, and the final version of the Spatial Skill Test consisted of 23 questions.

To improve transparency in the item selection process, Table 5 summarizes the sequential item-reduction procedure used during test development. Rather than relying on a single statistical criterion, decisions regarding item retention or removal were based on the combined evaluation of item difficulty (pj), item discrimination (rjx), lower–upper group comparisons, corrected item–total correlations, internal consistency (KR-20), expert judgments, and theoretical representation of the construct. This stepwise procedure reflects the iterative nature of instrument development and demonstrates that item retention decisions were guided by both statistical evidence and theoretical considerations.

**Table 5:** Summary of the sequential item reduction procedure.

Stage	Criterion	Items removed	Remaining items
Initial item pool	Expert review and content validity	–	35
Step 1	Item difficulty (pj) and item discrimination (rjx)	G2, G8	33
Step 2	Upper–lower group comparison	G6, İ3, İ11, Y3, Y11	28
Step 3	Corrected item–total correlation	G3, G7, İ1, İ9, Y10	23

Item elimination was conducted sequentially rather than simultaneously. Following each stage of item removal, all subsequent psychometric analyses were repeated using only the retained items from the previous stage. Therefore, decisions regarding item retention or removal were based on the cumulative evaluation of the updated psychometric evidence at each step rather than on the initial analyses.

As shown in Table 5, the development of the Spatial Skills Test followed a sequential item-reduction procedure in which each stage informed subsequent analyses. After each item removal stage, the remaining items were re-evaluated before proceeding to the next analysis. Consequently, decisions regarding item retention were based on the cumulative evaluation of multiple psychometric indicators together with theoretical considerations, rather than on a single statistical criterion. This iterative approach ensured that the final 23-item instrument maintained satisfactory psychometric properties while preserving adequate representation of the three spatial skill dimensions.

#### 3.3.6 Kuder–Richardson-20 (KR-20) reliability coefficient

The Kuder–Richardson-20 (KR-20) Reliability Coefficient is a coefficient used to evaluate the internal consistency of measurement instruments consisting of multiple-choice or true-false type dichotomous items. This coefficient is based on the relationship among the items that make up the test and the total variance of the test. KR-20 allows the interpretation of the reliability of the test by measuring the homogeneity among the items.

When the KR-20 value is 0.70 and above, the measurement instrument is generally considered reliable (Tavşancıl, 2014; Büyüköztürk, 2022). KR-20 is calculated based on the ratio of the variance of the entire test to the item variances and is particularly preferred in tests containing items of similar structure with fixed difficulty. A high KR-20 value indicates that the test items measure the same construct, and the test yields consistent results. Although the item-deleted reliability values are reported by IBM SPSS Statistics as “Cronbach’s Alpha if Item Deleted,” these estimates correspond to the KR-20 reliability framework because the present instrument consists entirely of dichotomously scored items (0 = incorrect, 1 = correct). Therefore, these values were used to examine the contribution of each item to the overall internal consistency of the instrument. The KR-20 reliability analysis results based on the item-total correlations of the 23-item Spatial Skill Test are presented in Table 6.

**Table 6:** KR-20 reliability analysis based on item-total correlations.

Item	Scale mean if item deleted	Scale variance if item deleted	Corrected item-total correlation	Cronbach’s alpha if item deleted
G1	14.06	21.803	0.258	0.825
G4	14.26	21.63	0.218	0.828
G5	14.2	21.442	0.278	0.825
G9	14.14	21.066	0.4	0.82
G10	14.13	21.585	0.272	0.825
G11	14.16	21.063	0.387	0.82
İ2	14.1	21.843	0.221	0.827
İ4	14.16	21.695	0.23	0.827
İ5	14.51	21.183	0.318	0.824
İ6	14.26	20.438	0.497	0.815
İ7	14.03	21.867	0.267	0.825
İ8	14.42	21.041	0.339	0.823
İ10	14.39	20.692	0.418	0.819
İ12	14.18	21.216	0.34	0.822
Y1	14.29	20.723	0.421	0.819
Y2	14.2	20.552	0.496	0.816
Y4	14.12	20.967	0.439	0.818
Y5	14.5	19.993	0.597	0.81
Y6	14.48	20.14	0.557	0.812
Y7	14.51	20.508	0.477	0.816
Y8	14.22	21.49	0.261	0.826
Y9	14.14	20.688	0.497	0.816
Y12	14.13	20.922	0.446	0.818
KR-20 coefficient: 0.827; N: 23				

Because all items were scored dichotomously (0 = incorrect, 1 = correct), the overall internal consistency was evaluated using the KR-20 coefficient. The “Cronbach’s Alpha if Item Deleted” values are automatically reported by IBM SPSS Statistics and are equivalent to KR-20 item-deleted reliability estimates for dichotomous items.

The results of the KR-20 reliability analysis indicated that the final 23-item Spatial Skills Test demonstrated good internal consistency. The KR-20 coefficient was calculated as 0.827, indicating that the instrument produced reliable and internally consistent scores. Although IBM SPSS Statistics reports the item-deleted reliability values under the heading “Cronbach’s Alpha if Item Deleted,” these estimates correspond to the KR-20 reliability framework because the instrument consists entirely of dichotomously scored items (0 = incorrect, 1 = correct). The corrected item-total correlation values of the items were mostly above 0.20, and only a few items yielded results close to the threshold value (e.g., for item G4, r = 0.218). Nevertheless, it is understood from the “Cronbach’s alpha if item deleted” values that these items did not reduce the overall reliability of the test, because no significant increase in the alpha coefficient was observed when any item was removed. This situation demonstrated that the comprehensive structure of the test could be preserved and applied, and each item provided a relevant contribution to the measured skills. As a result, in line with the results of the KR-20 test, it can be argued that the test was sufficient in terms of reliability in its current form (Büyüköztürk, 2022; Crocker and Algina, 2006). Although some retained items had relatively high correct-response rates, they were retained in the final form because they contributed to the representation of the target construct, did not reduce the test’s overall internal consistency, and supported the instrument’s accessibility for fourth-grade students.

### 3.4 Confirmatory factor analysis

**Figure 2:**
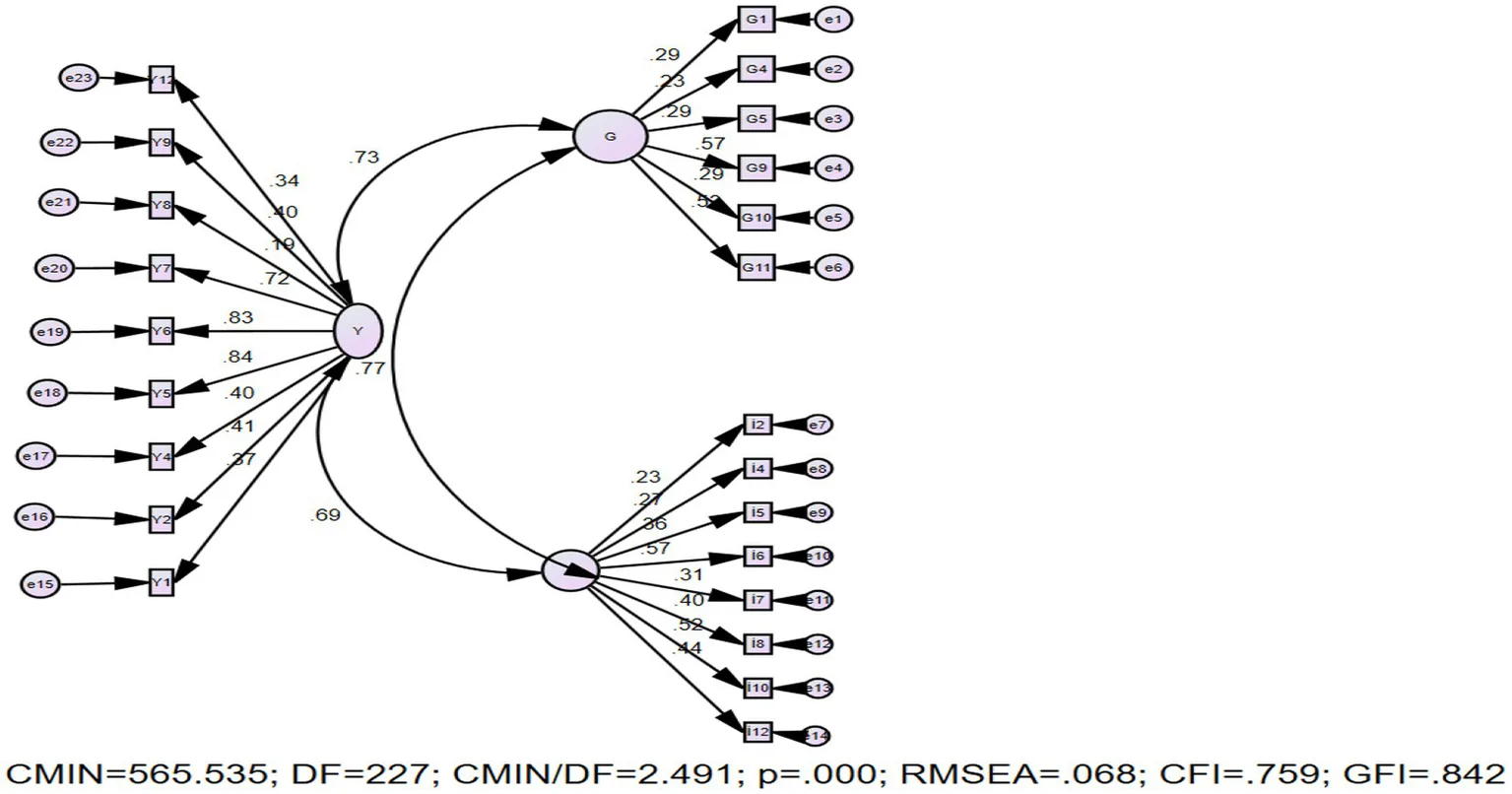
Path diagram of the spatial skill test.

The path diagram of the Spatial Skill Test is presented in Figure 2.

As shown in Figure 2, the Spatial Skills Test consisted of three dimensions and 23 items. The goodness-of-fit indices of the test are given in Table 6.

As shown in Figure 2, the Spatial Skills Test consisted of three dimensions and 23 items.

As seen in Table 7 and Figure 2, the model tested with CFA consisted of three latent variables (η1, η2, η3) and the observed variables representing them. Most of the standardized factor loadings were at acceptable levels, and some were above 0.80, which indicated that the items strongly represented the factors. In the examination of the goodness-of-fit indices, the values of *χ*^2^/df = 2.49 and RMSEA = 0.06 revealed that the model exhibited acceptable fit. The RMR value being as low as 0.01 showed that the errors were limited, while the GFI (0.84) and AGFI (0.80) values were supportive of a good fit, even if they were at the borderline. The CFI value remaining at 0.75 level indicated that the degree of comparative fit could be improved. Overall, the CFA results provided partial support for the proposed three-dimensional structure. Although *χ*^2^/df, RMSEA, and RMR/SRMR indicated acceptable absolute model fit, the CFI and GFI values remained below commonly recommended thresholds. Therefore, the construct validity evidence obtained from CFA should be interpreted with caution and considered together with the other psychometric findings presented in this study.

**Table 7:** Goodness-of-fit indices and reference ranges.

Indices	Excellent/good fit	Acceptable fit	Spatial skill test
*χ*^2^/Sd	0 ≤ *χ*^2^/df < 2	2 < *χ*^2^/df ≤ 5	2.49
RMSEA	0 ≤ RMSEA≤0.08	0.08 < RMSEA≤1.00	0.06
CFI	0.95 ≤ CFI ≤ 1.0	0.90 ≤ CFI < 0.95	0.75
GFI	0.95 ≤ GFI ≤ 1.0	0.90 ≤ GFI < 0.95	0.84
AGFI	0.90 ≤ AGFI≤1.0	0.85 ≤ AGFI<0.90	0.80
RMR	0 ≤ RMR ≤ 0.08	0.08 < RMR ≤ 1.00	0.01

Kline (2023) and Tabachnick and Fidell (2019).

No *post hoc* model modifications based on modification indices were performed. Although such modifications may improve overall model fit, they can also increase the risk of capitalizing on sample-specific characteristics. Therefore, the proposed three-factor structure was retained to preserve the construct’s theoretical integrity and the content validity established during instrument development.

### 3.5 Rasch model analysis

To complement the psychometric evidence obtained from Classical Test Theory and confirmatory factor analysis, a dichotomous Rasch model analysis was conducted to evaluate item fit, measurement precision, and item targeting. Table 8 presents the item difficulty estimates, along with the Infit and Outfit mean-square (MNSQ) statistics, for the final 23-item Spatial Skills Test.

**Table 8:** Rasch item fit statistics for the final 23-item spatial skills test.

Item	Difficulty (Logit)	Infit MNSQ	Outfit MNSQ
G1	−1.175	1.049	1.051
G4	0.162	1.221	1.456
G5	−0.207	1.108	1.274
G9	−0.569	0.956	0.933
G10	−0.650	1.088	1.003
G11	−0.431	0.983	0.843
İ2	−0.865	1.089	1.341
İ4	−0.431	1.148	1.304
İ5	1.510	1.134	1.217
İ6	0.145	0.888	0.812
İ7	−1.449	1.021	0.902
İ8	0.995	1.107	1.106
İ10	0.846	0.995	1.027
İ12	−0.318	1.037	1.088
Y1	0.332	0.986	0.941
Y2	−0.207	0.868	0.786
Y4	−0.692	0.902	0.745
Y5	1.439	0.766	0.671
Y6	1.352	0.830	0.734
Y7	1.510	0.943	0.895
Y8	−0.099	1.143	1.300
Y9	−0.549	0.848	0.688
Y12	−0.650	0.899	0.726

Acceptable item fit is generally indicated by Infit and Outfit mean-square values between 0.50 and 1.50.

As presented in Table 8, the item difficulty estimates ranged from −1.449 to 1.510 logits, indicating that the instrument included items representing a broad range of difficulty levels. The most difficult items were İ5 and Y7 (1.510 logits), whereas the easiest item was İ7 (−1.449 logits). Furthermore, the Infit mean-square values ranged from 0.766 to 1.221, and the Outfit mean-square values ranged from 0.671 to 1.456. Since all values were within the commonly accepted range of 0.50–1.50, none of the retained items demonstrated substantial misfit to the Rasch model. These findings indicate that all items contributed appropriately to measuring the intended construct and provided additional evidence supporting the internal construct validity of the Spatial Skills Test.

In addition to the item fit statistics, the Rasch model was used to evaluate the precision with which the instrument distinguished individuals with different levels of spatial ability. The results are presented in Table 9.

**Table 9:** Rasch person separation reliability.

Statistic	Value
Squared standard deviation (SSD)	1.50
Mean squared error (MSE)	0.321
Person separation reliability	0.786

As shown in Table 9, the person separation reliability coefficient was 0.786, indicating satisfactory measurement precision according to Rasch model standards. This finding suggests that the Spatial Skills Test is capable of distinguishing participants with different levels of spatial ability with an acceptable degree of reliability. Overall, the Rasch analysis complements the classical test theory findings by providing additional evidence for the psychometric adequacy of the developed instrument.

**Figure 3:**
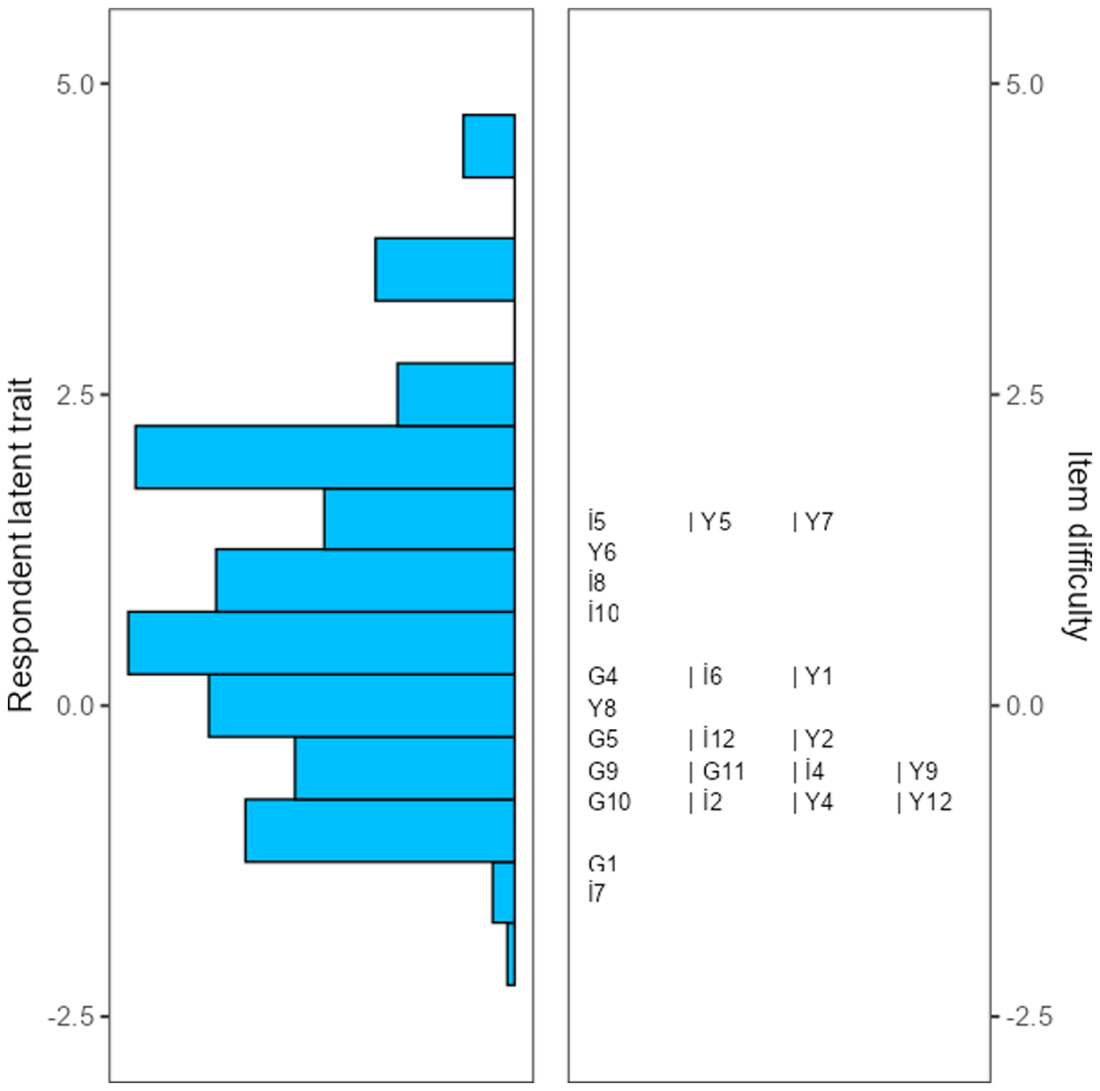
Wright map.

Figure 3 presents the Wright Map, illustrating the distribution of participants’ spatial ability estimates and the difficulty levels of the retained test items on the same latent continuum.

As illustrated in Figure 3, the retained items were distributed across a broad range of difficulty levels and were reasonably aligned with the distribution of participant abilities. Easier items were located in the lower region of the latent continuum, whereas more difficult items were positioned toward the upper region. This distribution suggests that the instrument provides appropriate measurement across varying levels of spatial ability and supports the adequacy of the item targeting for fourth-grade primary school students. Taken together, the Rasch model findings provide complementary psychometric evidence supporting the quality of the Spatial Skills Test beyond the classical test theory analyses and confirmatory factor analysis.

Overall, the findings from the Rasch model were consistent with those from classical test theory analyses. While the classical test theory analyses demonstrated satisfactory item discrimination and internal consistency, the Rasch analysis further confirmed that the retained items exhibited acceptable fit to the measurement model and satisfactory measurement precision. The convergence of evidence obtained from these complementary psychometric approaches provides stronger support for the validity and reliability of the Spatial Skills Test than relying on a single analytical method.

## 4 Conclusions, discussion, and recommendations

In this study, the psychometric properties of the Spatial Skill Test, which consisted of three dimensions and 23 items, were examined comprehensively. According to the KR-20 reliability coefficient, the internal consistency of the test was found to be high (*α* = 0.827), and this showed that the items contributed consistently to the construct they measured. Construct validity was examined using confirmatory factor analysis (CFA). Although the absolute fit indices suggested an acceptable fit, the incremental fit indices (particularly CFI and GFI) indicated that the proposed measurement model did not achieve an optimal fit. Therefore, the CFA findings should be interpreted as providing partial support for the hypothesized three-factor structure. Nevertheless, the consistency between the CFA results and the item analyses and reliability findings provides complementary evidence of the instrument’s psychometric adequacy. The overall fit indices of the model demonstrated that the construct provided an acceptable level of fit to the data (*χ*^2^/df = 2.49; RMSEA = 0.06; RMR = 0.01; GFI = 0.84; AGFI = 0.80; CFI = 0.75). The strong values of fundamental fit indices such as RMSEA and RMR show that the model has a reliable structure purified from residuals.

The findings indicate that the Spatial Skills Test provides a reliable and theoretically coherent assessment of fourth-grade students’ spatial skills. The KR-20 coefficient (0.827) demonstrates strong internal consistency, suggesting that the items consistently measure the intended construct. Likewise, the acceptable CFA fit indices support the hypothesized three-dimensional structure, providing empirical evidence that spatial visualization, spatial relations, and spatial orientation represent distinguishable yet related components of spatial skills in primary school students. To better understand the significance of these findings, they should be interpreted in relation to previous studies that have developed and validated spatial skills assessments across different educational levels.

A major strength of the present study is that the psychometric properties of the instrument were evaluated using multiple complementary approaches rather than relying on a single source of evidence. In addition to Classical Test Theory analyses, the factorial structure was examined through confirmatory factor analysis, and the measurement properties of the retained items were further supported using Rasch model analysis. By integrating Classical Test Theory, confirmatory factor analysis, and Rasch measurement within a single instrument development study, the present research provides more comprehensive psychometric evidence than many previous spatial skills assessments developed for primary school students.

Taken together, the findings obtained from Classical Test Theory, confirmatory factor analysis, and Rasch measurement provide complementary evidence supporting the validity and reliability of the Spatial Skills Test. The convergence of these independent psychometric approaches strengthens confidence in the instrument’s measurement quality and supports its use for assessing the spatial skills of fourth-grade primary school students in educational research and practice.

Spatial skill tests developed for university students (Açıkgül et al., 2023) and prospective teachers (Açıkgül-Fırat and Köksal, 2018; Şanlı, 2021), for students aged 11–14 at the middle school level (Göktepe-Yıldız and Özdemir, 2017; Akkaya-Yılmaz et al., 2022; Çokçalışkan, 2024), and for children at the primary school level (Kara et al., 2022; Demirel and Çetin, 2023) were structured in line with age-specific cognitive levels and measurement needs. In all these studies, the validity and reliability of the tests were examined with KR-20, Cronbach’s alpha, and test–retest analyses. For example, KR-20 values ranged between 0.63 (Demirel and Çetin, 2023) and 0.918 (Çokçalışkan, 2024). The test contents were mostly based on dimensions such as spatial relations, visualization, and orientation (Açıkgül-Fırat and Köksal, 2018; Göktepe-Yıldız and Özdemir, 2017), while some tests focused specifically on visual–spatial components such as mental rotation, integration, and paper folding (Dokumacı-Sütçü and Oral, 2019; Açıkgül-Fırat and Köksal, 2018; Demirel and Çetin, 2023). In contrast, the present study diverges from existing research by holistically addressing spatial visualization, spatial relations, and spatial orientation within a single measurement framework at the primary school level, thereby filling a structural gap specific to this age group.

The confirmation of the three-factor structure is theoretically meaningful because it supports previous conceptualizations suggesting that spatial skills are multidimensional rather than unitary (Clements, 1998; Contero et al., 2005; D'Oliveira, 2004). The CFA results therefore provide empirical support for applying these dimensions to primary school populations. This theoretical consistency may also explain why the instrument demonstrated satisfactory psychometric performance across multiple analyses. One possible explanation for the instrument’s satisfactory psychometric performance is that all items were developed within a clearly defined theoretical framework and specifically designed for fourth-grade learners. Rather than adapting instruments originally developed for adolescents or adults, the present study considered children’s developmental characteristics during item construction. This may explain the acceptable item discrimination, internal consistency, and construct validity observed in the study. These findings become more meaningful when interpreted alongside the psychometric evidence reported for previously developed spatial skills instruments.

The reliability coefficients obtained in the present study are comparable to those reported in previous spatial skills instruments developed for different educational levels (Göktepe-Yıldız and Özdemir, 2017; Akkaya-Yılmaz et al., 2022; Çokçalışkan, 2024). Although direct comparisons should be interpreted cautiously due to differences in participant characteristics and construct definitions, these findings indicate that the proposed instrument demonstrates psychometric quality comparable to established spatial assessment tools. While many of these instruments are psychometrically sound, with moderate difficulty and high discrimination indices (Şanlı, 2021; Kara et al., 2022), measurement efficacy varies by age and context. Indeed, Morawietz et al. (2024) demonstrated excellent reliability across diverse age groups, highlighting the importance of structuring spatial assessment tools in an age-sensitive manner.

However, the contribution of the present study extends beyond demonstrating comparable psychometric properties. While literature frequently emphasizes the use of concrete manipulatives—such as physical blocks, puzzles, and paper folding—in fostering spatial reasoning and spatial relation discovery (Casey et al., 2008; Hawes et al., 2022; Verdine et al., 2014), the unit-cube tasks in the present instrument were operationalized not as physical objects, but as *representational (pictorial) models* presented on a two-dimensional paper-and-pencil medium. Early experiences with concrete manipulative blocks (Clements, 1998) are transformed into mental representations within this assessment context, requiring primary school students to actively engage in mental rotation, integration, and perspective taking through unit-cube representations. Thus, the present study contributes to the theoretical literature by operationalizing spatial skills through three complementary dimensions (spatial visualization, spatial relations, and spatial orientation) within a mathematics education context. The findings support multidimensional perspectives of spatial cognition, demonstrating that these dimensions can be reliably assessed among primary school students using age-appropriate representational unit-cube tasks.

In addition to its theoretical contribution, the developed instrument holds practical implications for educational settings. By evaluating the transition from concrete manipulative experiences to representational spatial reasoning, the instrument assists teachers and researchers in identifying students’ specific strengths and weaknesses across spatial dimensions. Such diagnostic insights facilitate the design of targeted instructional interventions and enable more accurate evaluations of spatial training programs.

## 5 Limitations

The present study has several limitations that should be considered when interpreting the findings. First, although the proposed three-factor model demonstrated acceptable values for several absolute fit indices (e.g., χ^2^/df, RMSEA, and RMR), the incremental fit indices, particularly CFI and GFI, remained below commonly recommended thresholds. Therefore, the construct validity evidence obtained from the CFA should be interpreted together with the complementary evidence provided by the classical test theory and Rasch analyses rather than in isolation. Future research should re-examine the factorial structure using independent samples and cross-validation procedures.

Second, the study was conducted with fourth-grade students from a single province in the Black Sea Region of Türkiye. Consequently, the generalizability of the findings to students from different geographical regions, educational settings, and age groups should be interpreted with caution. Future validation studies involving more diverse and nationally representative samples are recommended.

Third, some items in the final form exhibited relatively high correct-response rates, suggesting that they were comparatively easy for the target population. Although these items were retained to preserve content coverage and developmental appropriateness, they may produce ceiling effects when administered to older students or higher-achieving groups. Future studies should therefore develop additional medium- and high-difficulty items and further evaluate item functioning across different populations.

Finally, the present instrument was intentionally limited to unit cube (block-based) item types to provide a developmentally appropriate and consistent assessment format for fourth-grade students. While this design enabled the assessment of spatial visualization, spatial relations, and spatial orientation within a common visual context, it does not encompass all aspects of spatial thinking, such as mental rotation, paper folding, perspective-taking, or map-based reasoning. Although the present study provides psychometric evidence based on Classical Test Theory, confirmatory factor analysis, and Rasch measurement, additional evidence should be established through test–retest reliability, measurement invariance across different groups, and item response theory (IRT) analyses using larger and more diverse samples.

## Data Availability

The datasets presented in this study can be found in online repositories. The names of the repository/repositories and accession number(s) can be found in the article/supplementary material.
